# Infliximab and Dexamethasone Attenuate the Ductular Reaction in Mice

**DOI:** 10.1038/srep36586

**Published:** 2016-11-08

**Authors:** Stefaan Verhulst, Jan Best, Wing-Kin Syn, Hendrik Reynaert, Karine H. Hellemans, Ali Canbay, Laurent Dolle, Leo A. van Grunsven

**Affiliations:** 1 Laboratory of Liver Cell Biology, Vrije Universiteit Brussel (VUB), Belgium; 2 Department of Gastroenterology and Hepatology, University Duisburg-Essen, Germany; 3 Section of Gastroenterology, Ralph H Johnson VAMC, Charleston, SC, USA; 4 Division of Gastroenterology and Hepatology, Medical University of South Carolina, Charleston, SC, USA; 5 Unit Diabetes Pathology and Therapy, VUB, Brussel, Belgium

## Abstract

Chronic hepatic injury is accompanied by a ductular response that is strongly correlated with disease severity and progression of fibrosis. To investigate whether anti-inflammatory drugs can modulate the ductular response, we treated mice suffering from a steatotic or cholestatic injury with anti-TNF-α antibodies (Infliximab) or glucocorticoids (Dexamethasone). We discovered that Dexamethasone and Infliximab can both modulate the adaptive remodeling of the biliary architecture that occurs upon liver injury and limit extracellular matrix deposition. Infliximab treatment, at least in these steatotic and cholestatic mouse models, is the safer approach since it does not increase liver injury, allows inflammation to take place but inhibits efficiently the ductular response and extracellular matrix deposition. Infliximab-based therapy could, thus, still be of importance in multiple chronic liver disorders that display a ductular response such as alcoholic liver disease or sclerosing cholangitis.

During chronic liver disease, progressive destruction of liver parenchyma and/or biliary structures, triggers a complex ductular reaction (or DR) located mainly within the periportal region, which ultimately constitutes the basic regenerative response^1^. Recent studies show that this DR is intimately associated with fibrosis and cancer^2^, and that a correlation exists between the extent of liver disease and the magnitude of the accompanying DR^3^,^4^.

The DR consists of innumerous bile ductular structures (or reactive-appearing duct-like cells) and intermediate ductal cells, both progenies of the hepatic stem/progenitor cells (HSPCs), with the latter residing in or near the canals of Hering^5^. Together with these biliary epithelial cell components (BECs), Kupffer cells (KCs), myofibroblasts (MFs), and the extracellular matrix (ECM) act in concert to correctly orchestrate the repopulation and reorganization of the damaged liver tissue^1^,^6^.

Tissue repair and regeneration of any organ in response to injury is invariably accompanied by macrophage infiltration^7^,^8^. Particularly, macrophages (or inflammatory monocytes) and their cytokine production play a major role in the DR upon chronic liver injury. The number of KCs increases in the liver prior to induction of HSPC proliferation and also spatially co-localize with HSPCs in a niche, suggesting that they may facilitate their recruitment/activation^4^,^9^,^10^ and that inflammation and epithelial repair are intrinsically linked^11^,^12^. Macrophages are an important source of soluble factors and cytokines such as tumor necrosis factor- alpha (TNF-α)^4^. TNF-α modulates a broad range of cellular functions including proliferation, survival, differentiation and apoptosis, and is considered an essential driver of the inflammatory immune response. Data from humans and animal models support a causative role for TNF-α in the pathogenesis of disorders such as alcoholic hepatitis, Crohn’s disease, rheumatoid arthritis, inflammatory bowel disease, and skin diseases, supporting the theoretical rationale for selective TNF inhibition as a beneficial treatment in those patients^13^,^14^,^15^,^16^. Among the TNF-α antagonists, Infliximab (IFX) is a chimeric monoclonal antibody that binds soluble TNF-α with high affinity and specificity to membrane-bound TNF-α on inflammatory cells and induces inflammatory cell apoptosis^13^,^15^,^17^. Besides anti-TNF-α agents, corticosteroids are widely prescribed anti-inflammatory compounds, which dampen the inflammatory response and are used to treat patients with allergies, asthma, and autoimmune diseases; dexamethasone (DEX) and prednisolone are the most commonly used glucocorticoids. Corticosteroids bind to steroid nuclear receptors, leading to the induction of anti-inflammatory factors and downregulation of pro-inflammatory pathways; in combination, these lead to the suppression of inflammation^18^.

Therapies that can stop the DR and the accompanying fibrosis are urgently needed. Therefore, we targeted the TNF-α axis by DEX or IFX and evaluated whether this would ameliorate liver injury and subsequent DR and fibrosis in the context of a steatotic (choline-deficient, ethionine-supplement diet: CDE) and a cholestatic (a 3, 5-diethoxycarbonyl-1, 4-dihydrocollidine-containing diet: DDC) disease model.

## Results

### Evaluation of liver injury upon CDE and DDC treatments

**Figure 1:**
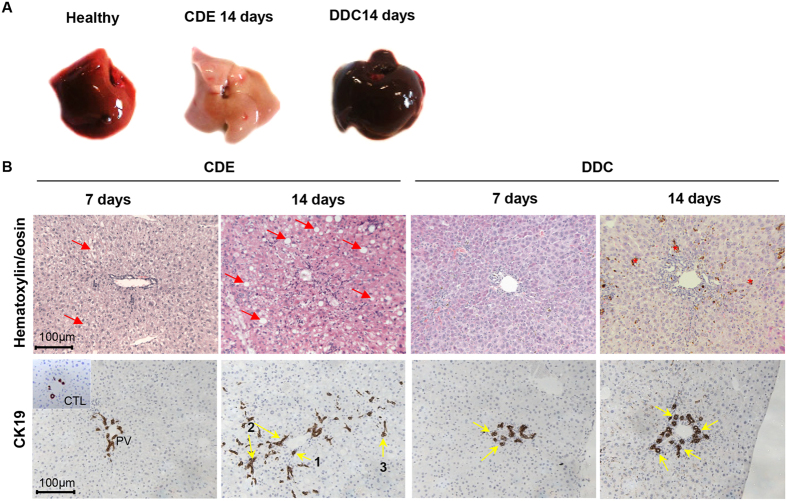
Histological evaluation of CDE- and DDC-treated livers. (**A**) Livers retrieved from unchallenged or mice-fed with a CDE or DDC diet for 14 days. Left lobes are divided for immunohistochemistry studies via embedding-paraffin processing. (**B**) Liver sections were stained with hematoxylin/eosin and CK19. After 7 days, CDE-treated livers accumulate fat (red arrows), which becomes more pronounced after 2 weeks of treatment, and small oval cells are appearing progressively, whereas porphyrin crystals appearing as brown pigments (red asterix) and stratification of bile ducts were the only striking features observed in livers of DDC-treated mice. In CDE livers, CK19^+^-cells are dramatically increased in number, forming arborescence inside the lobule. Those cells are organized in small clusters (1), or duct-like structures (2), or isolated (3) far from the PV area. DDC induces morphological alterations of the bile ducts (flattening of cholangiocytes, thickened multi-layered bile duct walls delineating a pseudo lumen) (yellow arrows). All images were taken in 20x original magnification.

In mice fed with a CDE diet (steatotic), the liver progressively turns to pale brown and has a harder texture than the bloody-red colour of unchallenged livers (Fig. 1A). At day 7, liver injury markers (ALT, AST, and LDH) were elevated while serum total-bilirubin progressively increased until day 14 (Fig. S1). CDE induces steatosis visible as vacuoles by HE staining (Fig. 1B red arrows). An expansion of CK19-positive cells (CK19^+^ cells) was already evident at day 7 of the CDE regime (Fig. 1B). Although the majority of CK19^+^-cells at day 7 were located in the periportal region (zone 1), later on, up to 20% CK19^+^-cells extended into the midlobular region (zone 2). By day 14, the number of CK19^+^-cells is dramatically increased in CDE-exposed livers, forming arborescence inside the lobule; cells are organized in small clusters, duct-like structures or isolated far from the portal vein area (Fig. 1B).

In DDC-treated mice (cholestasis), the liver turns to dark-brown (Fig. 1A) and shows a hard liver texture. ALT, AST and LDH at day 7 are increased and plateaued at day 14 (Fig. S1). Given that DDC is a porphyrinogenic drug, biliary obstruction was clearly apparent (Fig. 1B), and serum total-bilirubin drastically increased until day 14 (Fig. S1), resulting in a jaundiced state.

### Histological characterization of the ductular reaction induced by CDE and DDC diets

Besides the CK19^+^ cell expansion upon CDE damage, a DR was illustrated by a clear accumulation of F4/80^+^ cells, αSMA^+^ myofibroblasts, and an increase in cross-linked collagen (Sirius red^+^) and laminin deposition (Fig. S2) indicative of fibrogenesis. A similar outline of the emerging DR was observed with DDC feeding, but this process was more static in terms of the spatial localization of the DR. The CK19^+^ cells remained primarily in zone 1 with less spreading into the parenchyma compared to CDE treatment (Fig. S3). Accumulation of αSMA^+^ cells and deposition of laminin were also accompanied by an increase in cross-linked collagen and F4/80^+^ cells in the DDC-induced DR (Fig. S3). Our results confirm previous studies that distinguish the induction of cholestasis and steatosis in the DDC and CDE mice models, respectively.

### Impact of IFX and DEX on CDE-induced ductular reaction

**Figure 2:**
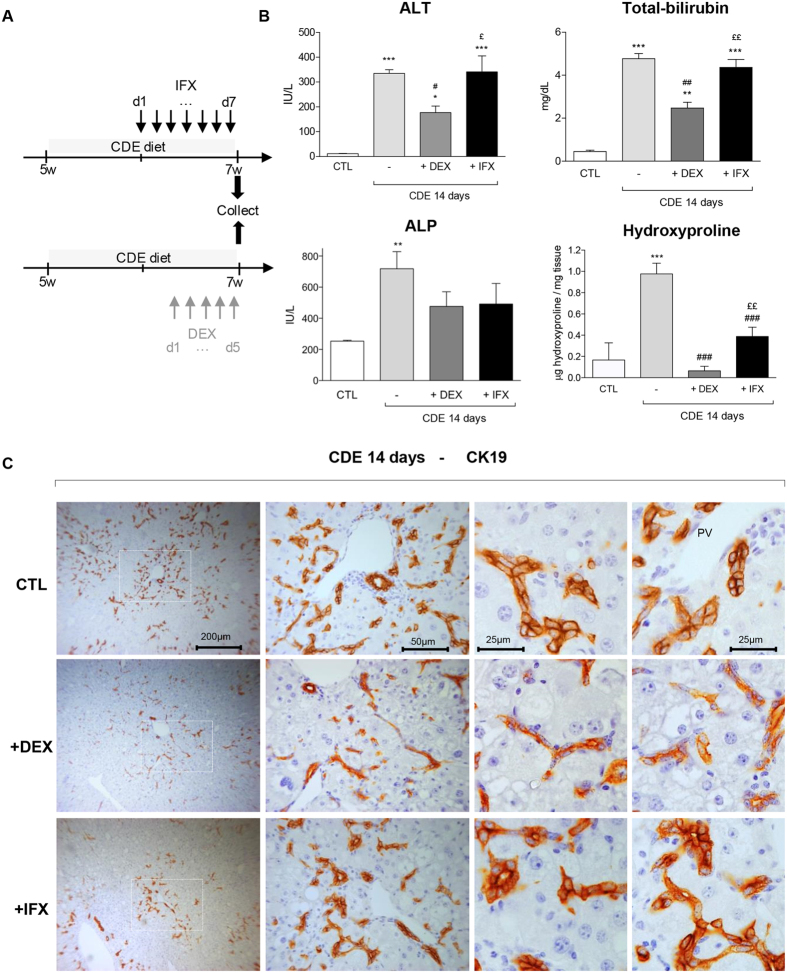
Impact of IFX and DEX on CDE diet mediated liver injury parameters and CK19^+^ cell distribution. (**A**) Experimental design: IFX was injected i.p daily from day 7 of CDE treatment whereas DEX was injected from day 9 onwards in a 14 day time course. (**B**) Quantification of ALT, ALP, and total-bilirubin in sera and hydroxyproline levels in livers from DDC-treated mice +/− DEX or IFX. (**C**) Representative pictures of immunohistochemistry for CK19 on CDE-treated livers with or without DEX or IFX are shown. The dotted windows in the left panels are magnified and presented in the middle panels. Higher magnifications of CK19^+^ cells are given in the two right panels. All images were taken in 20x original magnification.All data are mean (+/−SEM) for n = 5/group. *p < 0.05, **p < 0.01, and ***p < 0.001 difference versus CTL. ^#^p < 0.05 and ^##^p < 0.01 difference versus treated groups (CDE). ^£^p < 0.05 and ^££^p < 0.01 difference versus co-treated groups (+/−DEX or +/−IFX).

To mimic a potential clinical benefit of IFX and DEX on the DR, we choose to apply these drugs in a therapeutic setting for both models of liver injury (Figs 2A). Administration of IFX or DEX alone has no significant impact on the levels of biochemical parameters, neither on the colour and texture of the livers (Fig. S4), nor on the presence or the number of CK19^+^ and F4/80^+^ cells (Fig. S5). IFX or DEX never modulated the appetite of the treated mice; no particular change of fur was noticed nor was special behaviour detected.

Upon CDE treatment, the liver colour and texture were not modified by DEX or IFX (Fig. S6), nor was the blood volume harvested from those animals. When compared with vehicle-administration (CDE-treated), DEX administration significantly dimmed the high ALT levels normally observed with the CDE treatment, while IFX exhibited minimal effects (Fig. 2B). A similar inhibition on the total-bilirubin level was observed in mice receiving DEX, while in IFX-treated mice the bilurubin levels were unchanged (Fig. 2B). Both DEX and IFX decreased ALP levels (not significantly), indicative of less cholangiocytic injury. This suggests that only DEX injections significantly ameliorate the direct injury on hepatocytes induced by the CDE diet, but both DEX and IFX seem to affect the indirect injury on cholangiocytes.

**Figure 3:**
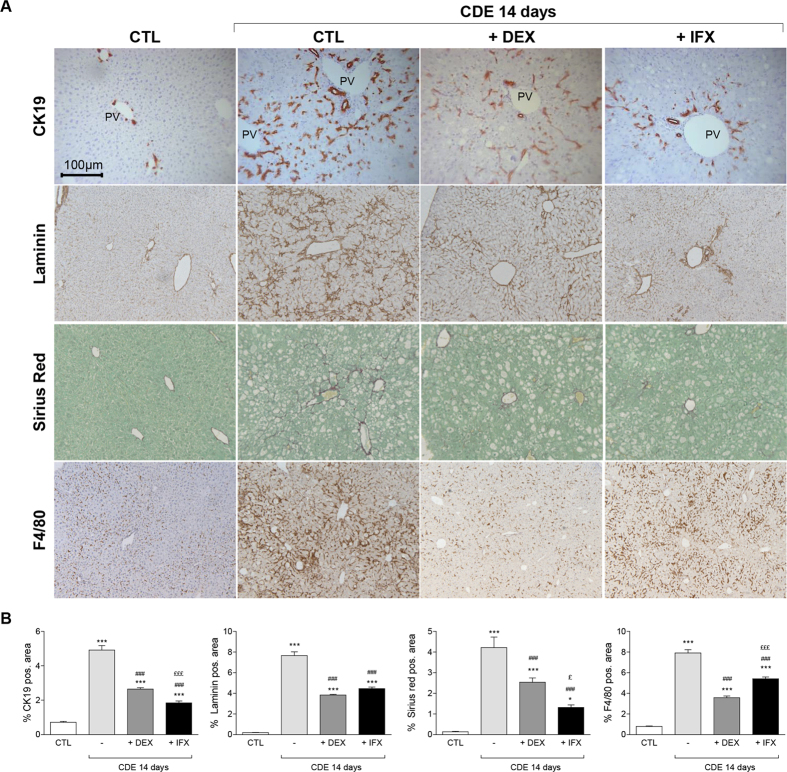
DEX and IFX attenuate the CDE-induced ductular reaction. (**A**) Liver sections of control animals and mice that were subjected to a 14 day CDE-diet in co-treatment with either IFX or DEX were stained with CK19, Laminin, Sirius red, and F4/80 antibodies. The pictures display representative photomicrograph. All images were taken in 20x original magnification. (**B**) Morphometric quantification. All data are mean (+/−SEM) for n = 5/group. *p < 0.05 and ***p < 0.001 difference versus CTL. ^###^p < 0.001 difference versus treated groups (CDE). ^£^p < 0.05 and ^£££^p < 0.001 difference versus co-treated groups (+/−DEX or +/−IFX). All images were taken in 20x original magnification.

Strikingly, both DEX and IFX blunted the DR when stained for CK19 (Fig. 2C). Whereas in CDE-treated livers CK19^+^ cells accumulated both in PV and interlobular zones, in DEX conditions, CK19^+^ cells were in the PV zone with fewer in its vicinity. On the contrary, IFX co-treatment disturbed CK19^+^ cell distribution, with CK19^+^ cells found predominantly in the PV zone (Fig. 2C). In the presence of DEX, the CK19^+^ cells are smaller in shape, elongated, and with a slight decrease of CK19 immunoreactivity present mainly as duct-like structures or individually but not in clusters as in control CDE livers (Fig. 1B). The overall presence of F4/80^+^ cells in the hepatic tissue was drastically inhibited by DEX but to a lesser extent by the use of IFX (Fig. 3). Earlier studies reported similar observations when using Clodronate-containing liposome to deplete the KCs^19^. While in CDE-injured livers, the CK19^+^ cell expansion was constantly associated with matrix deposition, both DEX and IFX co-treatment repressed laminin and collagen expression and deposition (Fig. 3, hydroxyproline Fig. 2B).

### Impact of IFX and DEX on DDC-induced ductular reaction

**Figure 4:**
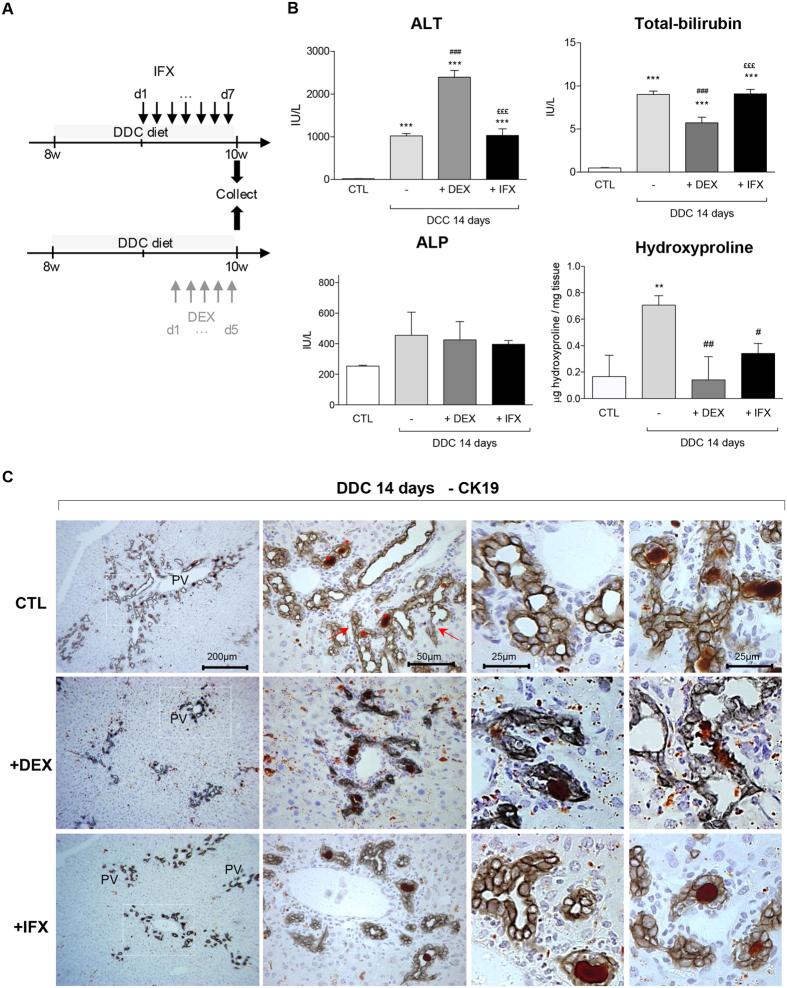
Impact of IFX and DEX on DDC diet mediated liver injury parameters and CK19^+^ cell distribution. (**A**) Experimental design: IFX was injected i.p daily from day 7 of CDE treatment whereas DEX was injected from day 9 onwards in a 14 day time course. (**B**) Quantification of ALT, ALP, and total-bilirubin in sera and hydroxyproline levels in livers from DDC-treated mice +/− DEX or IFX. (**C**) Representative immunohistochemistry images for CK19^+^ cells in DDC-treated livers with or without DEX or IFX are shown. The dotted windows in the left panels are magnified and presented in the middle panels. Higher magnifications of CK19^+^ cells are given in the two right panels. All data are mean (+/−SEM) for n = 5/group. ***p < 0.001 difference versus CTL, ^###^p < 0.001 difference versus treated groups (DDC). ^£££^p < 0.001 difference versus co-treated groups (+/−DEX or +/−IFX).

**Figure 5:**
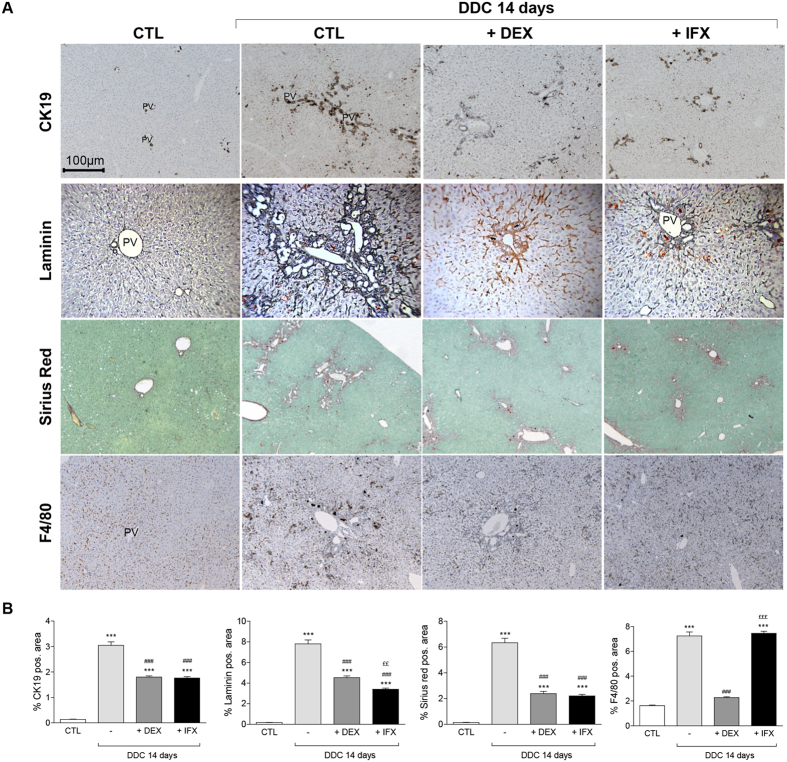
DEX and IFX attenuate the DDC-induced DR. (**A**) Liver sections of control animals and mice that were subjected to a 14 day DDC-diet in co-treatment with either IFX or DEX were stained with CK19, Laminin, Sirius red, and F4/80 antibodies. (**B**) Morphometric quantification. All data are mean (+/−SEM) for n = 5/group. ***p < 0.001 difference versus CTL. ^###^p < 0.001 difference versus treated groups (DDC). ^££^p < 0.01, and ^£££^p < 0.001 difference versus co-treated groups (+/−DEX or +/−IFX). All images were taken in 20x original magnification.

Upon DDC treatment, the liver colour was not modified by DEX or IFX (Fig. S6) neither was the texture. However, higher levels of ALT indicated that the hepatic injury is two-fold more severe in the DDC/DEX-co-treated mice than in mice treated only with DDC diet, while co-administration of IFX has no impact on ALT levels (Fig. 4B). Interestingly, while both IFX and DEX do not affect the accumulation of porphyrin in DDC-fed livers, only DEX co-administration reduces the levels of bilirubin in serum of co-treated animals. DDC treatment leads to a mild increase in ALP which is not affected by co-treatment of DEX or IFX (Fig. 4B). Co-treatments with DEX or IFX reduce the ductular response as evidenced by CK19 stainings (Fig. 4C); DEX co-treatment results in significantly altered architecture of the biliary system, impeding the pseudo-tubular structure, while IFX did not have this effect. The attenuation of CK19-positivity was also associated with a drastic inhibition of ECM deposition (Fig. 5 and hydroxyproline Fig. 4B). As expected, DEX affected the amount of F4/80^+^ cells while IFX seems to affect the distribution of the F4/80^+^ but not the amount. Morphometric and mRNA analyses confirmed these observations (Figs 5B and S7). Altogether, our data show that DEX and IFX reduce ECM deposition and attenuate the DR.

### IFX and DEX differentially affect the spatial distribution of F4/80^+^ cells upon CDE and DDC injury

#### Spatial distribution of F4/80-positive cells in livers damaged by CDE and DDC diets.

Since the difference in amount and distribution of F4/80^+^ was striking when comparing CDE and DDC diets, we further investigated their localization and quantity in the different dietary setting. In unchallenged livers, single F4/80^+^ cells with elongated cytoplasm and small nuclei were generally located in the periportal and midlobular regions, sparing the centrilobular regions (Fig. S8). After 7 days of CDE, KCs appeared rounder and enlarged, and were arranged in clusters around the bile ducts, but the majority was found farther away from the portal area. By day 14, F4/80^+^ cells had formed interconnected networks, which were equally concentrated around the centrilobular and portal veins (Fig. S8A). When quantified, this shift in F4/80^+^ cell distribution between the CV and PV is even more striking (Fig. S8B). In contrary, upon DDC treatment, F4/80^+^ cell accumulation was slower, less extensive, and almost exclusively located within the portal fields or in close proximity to porphyrin crystals (Fig. S8).

#### Impact of DEX and IFX on the spatial distribution of F4/80-positive cells in mice treated with CDE and DDC diets

**Figure 6:**
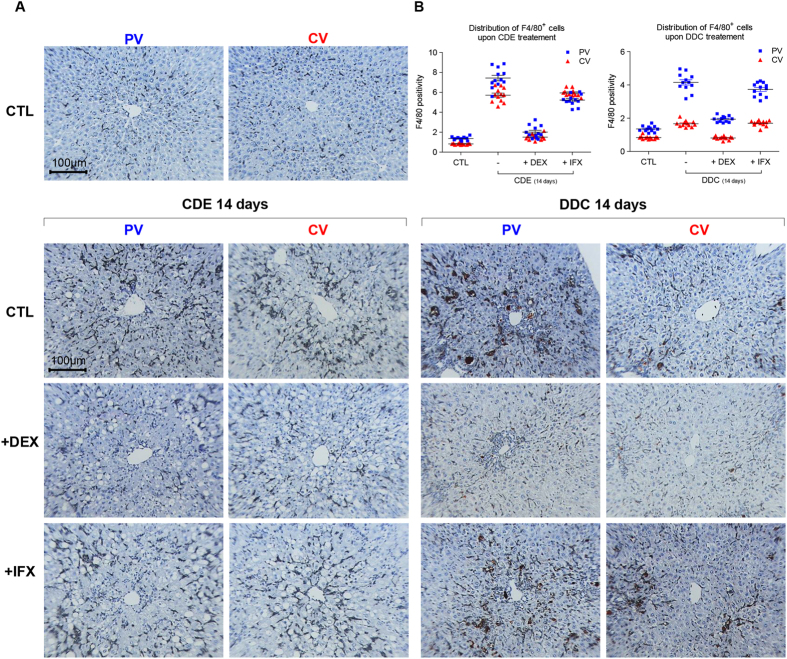
DEX and IFX modulate the recruitment of F4/80^+^ cells in both CDE- and DDC- models. (**A**) Healthy mice or subjected to a period of 14 day CDE-and DDC-diet in presence or absence of DEX or IFX were analyzed for spatial distribution of F4/80^+^ cells. Livers sections were stained with F4/80 antibody and counterstained with hematoxylin. The images display representative photomicrograph from portal- and central vein areas. All images were taken in 20x original magnification. (**B**) Morphometric quantification of stainings performed in (**A**). All images were taken in 20x original magnification.

Analysis of the F4/80^+^ cell distribution in both models of liver injury, with or without DEX or IFX, showed that F4/80^+^ cells normally gather massively around the portal vein after 14 days of CDE diet, while such distribution was virtually absent in DEX-administered mice and more comparable to unchallenged livers (Fig. 6A). While F4/80^+^ cells were confined to midlobular regions around the PV in IFX-administered mice, we also noticed that these cells highly accumulated in CV areas compared to the DEX-administrated mice. Upon cholestatic damage, the F4/80^+^ cell distribution in co-treated livers by IFX seemed to be similar to that of DDC treatment alone, while DEX exhibits a drastic inhibitory effect on the KCs (Fig. 6B). To gain some mechanistic insight, we investigated the impact of these two treatments on the expression of genes involved in marcrophage recruitment and BEC proliferation (or differentiation) in the DDC- and CDE-treated mice; mRNA levels of the pro-inflammatory cytokines Tnf-α and Il6 and the monocyte chemoattractant protein-1 (Mcp1) (Fig. S9) were decreased by DEX and IFX independently of the liver injury model. Strinkingly, compared to the CDE model, only very low levels of Mcp-1 mRNA levels were detected in the DDC-diet conditions. In contrast, mRNA levels of Tnfr-1 and -2, Jag-1 Notch-1 and -2, IFNɣ and Tweak (Tumour necrosis factor-like weak inducer of apoptosis) were only influenced by DEX. This confirms the more selective action of IFX and suggests that at least in the CDE diet, down regulation of Mcp-1 could be responsible for the reduced amount of F4/80^+^ cells in DEX and IFX-treated animals.

### DEX and IFX attenuate the adaptive remodeling of the biliary architecture and modulate the proliferative status of ductular cells

While DEX and IFX blunted the increase in CK19^+^ cells, a subtle difference was visible on the remodeling of the biliary architecture by these 2 drugs (Figs 3A and 5A). To investigate this further, we quantified the distribution of the CK19^+^-area close to the portal vein and to its vicinity upon CDE treatment in presence or absence of DEX or IFX (see Fig. S10). Notably, while IFX has a stronger inhibitory effect on the CK19^+^-BEC expansion found in the PV’s vicinity, DEX inhibits more drastically the expansion of the CK19^+^ cells from the PV’s vicinity (Fig. S10). Our results confirm earlier data showing that in response to different injuries the plasticity of the biliary tree is remodelled accordingly^20^,^21^, and suggest that the use of IFX and DEX may have an unprecedented impact on the flexibility of this hepatobiliary system. Contrarily to CDE, in DDC settings the remodeling of the biliary architecture was obvious and was therefore not quantified.

**Figure 7:**
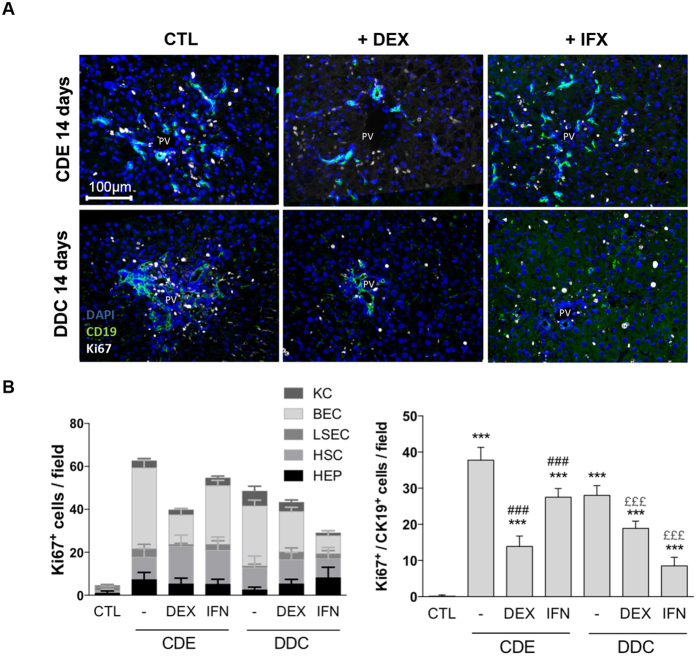
DEX and IFX influence the proliferative status of the DR in CDE- and DDC-treated livers. (**A**) Mice received CDE or DDC dietary regimes for 14 days to induce liver injury. Representative pictures of the detection of CK19/Ki-67 double positive cells in presence or absence of DEX or IFX are shown and (**B**) Quantification of the amount of Ki-67 positive cells per hepatic cell type per field: hepatocytes (HEP, Albumin^+^), HSC (Desmin^+^); LSEC (Lyve1^+^), KC (F4/80^+^), BEC (CK19^+^). Right graph: representation of only Ki-67/CK19 double positive cells per field. PV = portal vein. All data are mean for n = 5/group. ***p < 0.001 difference versus CTL. ^###^p < 0.001 difference versus treated groups (DDC or CDE). ^£££^p < 0.001 difference versus co-treated groups (+/−DEX or +/−IFX). All images were taken at 20x original magnification.

Finally, we hypothesized that at least part of the observed effects of IFX and DEX could be due to an inhibition of BEC, HSC and F4/80^+^ cell proliferation under these experimental conditions. Overall, both dietary regimens predominantly resulted in an increase in proliferating Ki-67^+^/Desmin^+^-HSCs (Fig. S11) and Ki-67^+^/CK19^+^-BECs (Fig. 7). Very little proliferation was observed for hepatocytes, LSECs and Kupffer cells (Fig. S11). While DEX decreased the number of Ki-67^+^ BECs by 63% in CDE- and by 40% in DDC-treated animals, IFX treatment inhibited the amount of Ki-67^+^ BECs by 27% in CDE- and by 70% in DDC-treated animals. At the same time, DEX and IFX treatment did not significantly affect the number of proliferating HSCs, suggesting that the major impact on fibrogenesis by these two treatments is not due to inhibition of HSC proliferation (Fig. S11).

## Discussion

In this study, we evaluated whether targeting the TNF-α axis may be useful in ameliorating liver injury parameters and the DR in the context of a steatotic and a cholestatic model of liver disease. Our results clearly show that (i) TNF-α is essential for the adequate development of the DR in two liver injury models; (ii) in the steatotic CDE model, only DEX is able to reduce the liver injury, while in the cholestatic model DEX worsens the injury; (iii) in the CDE model, the migration of CK19^+^ cells and the PV localization of F4/80^+^ cells is dependent on TNF-α. We conclude that both DEX and IFX can modulate the DR and inhibit extracellular matrix deposition in a cholestatic and a steatotic mouse model of liver injury. Overall, our findings are encouraging and indicate that IFX treatment, at least in these steatotic and cholestatic mouse models, is the safer approach since it does not increase liver injury, allows inflammation to take place and ameliorates hepatic proliferation, but efficiently inhibits the DR and extracellular matrix deposition.

Previous studies have shown that drastic modulation of the inflammatory response is possible by depleting macrophages from the organ of interest by means of nanoparticles, like clodronate-containing liposomes^22^. However, KCs have a biphasic role in liver injury^8^,^23^,^24^ and depleting them at different stages of the DR may make it difficult to predict the outcome. We opted for the systematic administration of DEX. A drawback of this approach is the systemic immunosuppression which makes it also technically difficult to determine which cells have been targeted. Recent studies using models of acute and chronic experimental injury have shown that DEX-loaded liposomes compared to free DEX may be a more efficient tool not only to target macrophages but also to modulate their inflammatory cytokine responses and their migratory properties^25^,^26^. In our experiments, DEX did affect ALT levels in DDC-treated mice, nonetheless it still dimished bilirubin levels and decreased collagen and laminin deposition and CK19^+^ cell expansions. This could mean that DEX encapsulation could reduce the effect on cells that are otherwise sensitive to free DEX, which then could reduce its positive benefit on fibrosis and CK19 expansion^25^.

After *i.p.* injection, IFX and DEX enter in the peritoneum, are taken up by the mesenteric drainage system, and then get into the systemic circulation mostly through the hepatic portal system. We consent that our results, although significant in describing the role of inflammatory cells in these models, do not exclude whether those effects are linked to collateral effects, and more particularly on the gut microbiota. Since TNF-α is an important tight-junction regulator of the intestine barrier, injecting DEX or IFX *i.p*. could also impede the cell signaling at this place. Further studies are thus required to explore whether there is less dysbiosis in co-treated animals.

Lately, substantial evidence demonstrates that sensitization to biological drugs is an important clinical problem. The emergence of “nonresponse,” “loss of response,” or “adverse events” to a certain therapy complicates the management of patients in remission. Particularly with TNF-α agents, loss of response to therapy has been associated to the presence of antibodies that bind to IFX itself, impairing its function and therefore influencing the IFX therapeutic efficacy^27^. Evidence shows that higher IFX concentrations are associated with greater clinical efficacy in patients with Crohn’s disease and that both presences of antibodies to IFX and low IFX concentrations are associated with worse clinical outcomes^28^. Consequently, to make a definitive statement on the safety of IFX in our experimental settings, future research should evaluate the optimal IFX dosing. We noticed in our preliminary experiments that dosage and frequency of IFX treatment has a big impact on the outcome; for example 1, 2, or 3 i.p injections of IFX at the beginning or spread over the last week of the liver injury were inadequate to observe a decline of the DR (not shown). However, even with daily injection of IFX, no mortality was seen in the concerned animal groups regardless of the nature of the injury, illustrating that the safety profile of IFX is acceptable. Nevertheless, to better judge potential toxicity of DEX and IFX in future studies, other organs should be investigated as well.

Another aspect we did not consider in this study, is the use of other TNF-α-blocking agents such as adalimumab, golimumab^17^ or etanercept^29^ to determine whether one of these clinically approved monoclonal antibodies has a better efficiency and efficacy to abolish the DR and fibrosis. All these TNF-α blockers contain the IgG1 Fc portion, which on one hand improves the *in vivo* half-life, but on the other hand leads to unwanted effector-mediated cytotoxic effects. Recently, a new anti-TNF-α agent named TNFR-hyFc (TNFR with a hybrid Fc) showed a higher neutralizing activity and even a better protective effect in an arthritis model compared to Etanercept administration^30^. Comparison of these blockers in different liver injury models would determine if our first choice on IFX was a good one or whether blockers like TNFR-hyFc are more adequate to attenuate the DR from one particular liver disease. Given their susceptibility to physical and chemical degradation processes, anti-TNF-α agents would require the development of stable formulations and specific delivery strategies should be developed to reduce the risk of side effects caused by prolonged presence of anti-TNF-α blockers^31^. In this study, we only exposed mice for a short term to DEX and IFX, which already resulted in a strongly reduced DR. We do not exclude that longer treatment with these agents in a chronically diseased liver that might need the regenerative capacity of the DR for hepatocyte repopulation could be harmful. However, the only mouse model in which CK19^+^ cells massively contributed to liver regeneration is the recently reported AhCre^+^ Mdm2^flox/flox^ mouse, in which Mdm2 inactivation leads to complete senescence of hepatocytes and the activation of CK19^+^ HSPCs is indeed necessary for survival and complete functional liver reconstitution^32^. In the used DDC and CDE models, no or only a minor contribution of HSPCs to liver reconstitution could be demonstrated^33^,^34^, thereby miminizing the risk of an adverse affect of long-term DR inhibition in these models.

We gained some insight into the mechanisms by which DEX or IFX influence the DR and fibrogenesis in these two liver injury models. Generally, when compared to IFX, DEX administration influences many more signaling pathways as shown by the inhibition of Jag1, Notch1 and -2, as well as Tnfα and Tnfr1 and -2 mRNA expression which were not, or much less, affected by IFX. Both treatments resulted in the downregulation of Mcp1 and Il6 in the CDE setting (Fig. S9), which coincided with less recruitment of F4/80^+^ cells. This was not the case for the DDC diet, in which only DEX resulted in the lesser expression of Mcp1, while IFX did not influence Mcp1 mRNA levels and did not have an impact on the amount of F4/80^+^ cells in the DDC-treated livers. However, the levels of Mcp1 mRNA were not increased by DDC while CDE treatment results in a 50 fold increase of Mcp1 mRNA levels, suggesting that probably in DDC-treated mice Mcp1 is not responsible for the macrophage infiltration. Neither IFX, nor DEX, affected the proliferation of F4/80^+^ cells suggesting that the difference in the F4/80^+^ cells is mainly due to a difference in recruitment of macrophages. A possible mediator in the effect of DEX on these injury models is Tweak, a known mitogen for HSPCs that acts through the FN14 receptor^35^ and is necessary for liver fibrosis following chronic CCl_4_ injury^36^. Tweak mRNA levels are decreased by DEX in both injury models, which could explain the lesser DR and fibrogenesis when DEX is used (Fig. S9). Of note, recent *in vitro* experiments with HSCs showed that Tweak could affect proliferation and not activation of HSCs^36^ while in our experiments HSC is not affected even though DEX decreased Tweak mRNA levels. Moreover, the DEX mediated inhibition of these signaling pathways apparently outperforms the potential downstream negative effects of an increased hepatocytic injury (higher ALT values) in the co-treated DEX/DDC animals (Figs 4 and 5). Thus, mechanistically we can only state that, DEX affects many signaling pathways including Notch, Tnfα and Tweak signaling while IFX is less disruptive, but also leads to a similar diminishment of DR and fibrosis. IFX and DEX both lead to less macrophage recruitment as observed in the CDE diet and perhaps directly affect survival of HSCs by inhibition of TNFα signaling^37^; IFX directly by acting on TNFα, and both DEX and IFX by downregulation of Tnfα mRNA levels (Fig. S9). Clearly, more studies using cell type specific knock-out mice of these signaling mediators are needed to unravel the exact mechanisms involved in the development of the DR and fibrogensis in these two mouse models of liver injury and to further explain the impact DEX and IFX have on liver disease progression.

Targeting the DR as an approach to improve regeneration or at least inhibit fibrogenesis is not novel. Knight *et al*. showed, that at least in mice the DR may drive fibrogenesis during chronic liver injury^9^,^38^. A recent report demonstrated that cues that regulate the DR also influence fibrosis progression; blocking TWEAK caused a reduction in both HSPC numbers and collagen deposition in a partial hepatectomy mouse model of fibrotic livers^39^. Studies on NAFLD patients and *in vitro* studies with BECs and HSCs suggested that CK19^+^ ductules can influence the progressive portal and bridging fibrosis in NAFLD^40^. Finally, there is an important DR in advanced alcoholic liver disease patients^41^,^42^ of which the extent is correlated with the severity of the disease^43^,^44^. Based on our data in both models of liver disease, one could suggest that in alcoholic liver disease patients with important DR, perhaps IFX treatment could have beneficial effects. In fact, clinical trials with alcoholic hepatitis patients treated with IFX alone^45^,^46^ or in combination with prednisone^47^ were promising. Unfortunately, the results of two large randomized controlled trials testing IFX^48^ or etanercept (soluble TNF receptor)^49^ were disappointing and reported high rates of infection, perhaps due to the sequential infusions, the major difference with the succesful single dose IFX only trials^45^,^46^. Currently, there are no large ongoing studies of targeted treatment for alcoholic hepatitis based on IFX^50^. Nonetheless, we believe that our findings could be of importance for the further development of drugs for multiple chronic liver disorders that frequently display a ductular response such as patients with alcoholic liver disease or sclerosing cholangitis.

## Material and Methods

### Animals and Study design

All methods, experimental protocols and animal experiments were performed according to the to European Guidelines for the Care and Use of Laboratory Animals and were approved by the Ethical Committee of Animal Experimentation of the Vrije Universiteit Brussel (VUB, Belgium: Project # 09-13-212-1). Male C57Bl/6J mice (Charles River Laboratories, France) were maintained at temperature, humidity and light-dark cycle (with a day light period from 07.00 a.m. to 19.00 p.m.) controlled conditions in all experiments. Five mice of 4- and 7-week-old of age were allocated per cage and allowed food and water *ad libitum*. After 1 week of acclimatization, mice were randomly assigned to different experimental groups. In one group, 8-week-old mice were fed with a 3, 5-diethioxycarbonyl-1,4-dihydrocollidine (0, 1% wt/wt) (DDC, C14H21NO4, Sigma-Aldrich, 137030, Bornem, Belgium) containing diet (C1000, Altromin, Lage, Germany) for 7 and 14 days to induce a cholangiocytic injury^51^. In another group, 5-week-old mice were fed with a diet deficient in choline (MP Biomedical, Irvine, USA) supplemented with 0, 15% (wt/vol) ethionine (Sigma-Aldrich, E5139) in drinking water (CDE) for 7 and 14 days to induce a hepatocyte injury^52^. Untreated, age-, and sex-matched mice were used as control. Food and water were changed every 3 days and ethionine-containing water bottles were protected from light.

In a first set of experiments, mice were treated with dexamethasone (DEX, 1mg/kg, Sigma) by a daily *i.p.* injection during the last 5 consecutive days of 2 weeks DDC or CDE-treatment. In a second set of experiments, after 1 week of dietary treatment, the mice were *i.p.*-injected daily with Infliximab (IFX or Remicade^TM^, MSD, Belgium, 25μg/g) for an additional 7 days after the beginning of the treatment. IFX and DEX were diluted in sterile PBS and then filtered through 0.22 μm filter prior to injection. Control group animals received saline injections. Regardless of the treatment, males with same age by group were used and 100 μL maximum of solution was injected.

See Supplementary Information for other standard protocols, materials and products used in this study.

## Additional Information

**How to cite this article**: Verhulst, S. *et al*. Infliximab and Dexamethasone Attenuate the Ductular Reaction in Mice. *Sci. Rep.* **6**, 36586; doi: 10.1038/srep36586 (2016).

**Publisher’s note:** Springer Nature remains neutral with regard to jurisdictional claims in published maps and institutional affiliations.

## Supplementary Material

Supplementary Information
