# Supplementary material for: Infliximab and Dexamethasone Attenuate the Ductular Reaction in Mice
Source: Sci Rep. 2016 Nov 8;6:36586. doi: 10.1038/srep36586 (PMC5100545; doi:10.1038/srep36586)
Supplement: Supplementary Information [file srep36586-s1.pdf]

Infliximab and Dexamethasone Attenuate the Ductular Reaction in Mice

Verhulst Stefaan, Best Jan, Syn Wing-Kin, Reynaert Hendrik, Hellemans Karine H, Canbay Ali, Dollé Laurent[‡*] and van Grunsven Leo A[‡*]

Supplemental methods

Sample collection

For all experiments, after 2 weeks of CDE or DDC diet, animals were anaesthetized with Pentobarbital anaesthesia (Nembutal^{TM}, Ceva) intraperitoneally, blood samples were withdrawn by inferior vena cava puncture, and sera were obtained after centrifugation (+4°C, 10.000 rpm, 10min.) and analyzed directly for biochemical markers of liver dysfunction. The gross anatomy of each liver was observed, as well as its colour and textures were noticed. Regardless of the treatment, liver preparation and processing were consistently prioritized: left lobes were systematically selected and fixed in 4% formalin for further immuhistological analyses or frozen in OCT (Labonord, Templemars, France) for additional histological analyses or PCR analysis, and the remaining lobes of the liver were snap frozen in liquid nitrogen and stored at -80°C until their further use.

Serum Biochemistry

The severity of the injury was assessed by detecting levels of different clinical relevant parameters, such as ALT, AST, total-bilirubin and -protein, ALB and LDH using a clinical chemistry module (Spotchem EZ SP-4430, Axon Lab AG, Stuttgart, Germany).

Hydroxyproline assay

The amount of hydroxyproline in total livers was performed using the method of Kesava Reddy G, and Enwemeka CS. A simplified method for the analysis of hydroxyproline in biological tissues. Clinical Biochemistry. 1996;29(3):225-9. Briefly, 100mg total liver was

hydrolyzed in HCl(6N) and incubated with Chloramine T (2.5mM) and Ehrlich's reagent. The amount of hydroxyproline content was measured using photometric analysis with an absorption of 570nm.

Immunohistochemistry and Immunofluorescence

For immunohistochemical detections, 4 µm liver sections were dewaxed and rehydrated in baths of graded alcohol. Endogenous peroxidase was blocked by the dual endogenous enzyme block solution (Dako, S2003) for 20 minutes. For antigen retrieval, slides were submitted to different conditions depending on the nature of the antibody (see Table S1 for the right conditions used). The block serum-free ready-to-use solution (Dako, X0909) was used as blocking solution (for 45min @RT) then sections were incubated with specific primary antibodies (summarized in Table S2). Detection was performed using anti-rat peroxidase-coupled secondary antibody (Dako, E0468), anti-goat or anti-rabbit Envision+ System-poly-HRP labelled polymer (Dako, K4003). Peroxidase activity was revealed by diaminobenzindine (Sigma, D5637) in which nickel and cobalt solutions (1% ready-to-use solutions) were added to DAB solutions to get a black instead of brown colour (in DDC dietary regime, porphyrin crystals appear brown on liver tissue). Slides were counterstained with Harris hematoxylin modified solution (Sigma, HHS16) or by Methyl Green (Vector Labs, H-3402) according to the manufacturer instructions.

Sirius Red staining

To assess collagen deposition, 4µm paraffin sections were dewaxed, rehydrated, and fixed with a mixture of direct red 80 (Sigma, 365548), Fast green FCF (Sigma, F7258) and saturated picric acid (Sigma, 925) for 45min at RT and light-protected. Slides were washed with water and dehydrated in graded alcohols and then mounted.

Morphometric analysis

To quantify immunohistochemistry and immunofluorescence stainings, 10-20 representative pictures using a 20x magnification were taken from each mouse and analyzed using ImageJ. Area percentages (immunohistochemistry) or number of double positive cells (immunohistofluorescence) per field were imported into Graphpad Prism for statistical analysis. Ki-67 positive cells were changed from red (594nm) to white using ImageJ for better visualization and to facilitate the counting. For CK19/Ki-67 stainings, consecutive sections were used and only clearly superimposable sections were used for quantification.

RNA extraction, reverse-transcription and PCR

Total RNA from liver tissue was extracted using Trizol (Invitrogen, Eugene, OR) and RNeasy kits, respectively (Qiagen, Hilden, Germany). Total RNA was converted to cDNA by reverse-transcription using the Revert Aid Kit (ThermoFisher Scientific, St. Leon-Rot, Germany). The RT reaction was performed at 25 °C for ten minutes followed by 30 minutes at 50 °C. For quantitative real-time polymerase chain reaction (qPCR), GoTaq QPCR Master Mix with BRYTE green (Promega, Madison, WI) was used, subjected to qPCR in a 7500 real time PCR system and analyzed using System SDS software v2.0.5 (Applied Biosystems) using gapdh for normalization. Gapdh mRNA was chosen as an invariant standard house-keeping gene (among 18S, β-actin, Hmbs, Hprt1 and Sdha genes using BestKeeper; data not shown). Fold change differences between samples were determined using the comparative Ct (δδCt) method. The expression level of different targets, relative to gapdh, was given by 2-δδCt. Gene-specific primers produced by Integrated DNA Technologies (Leuven, Belgium) are listed in Table S3.

Statistics

All data were expressed as mean ±SEM. Statistical analysis was performed using Student's test or one-way ANOVA (with Tukey's multiple comparison test). All analysis was conducted using Graph-Pad Prism software (GraphPad Software). Statistically significant differences were considered at $p<0.05$.

Supplemental results.

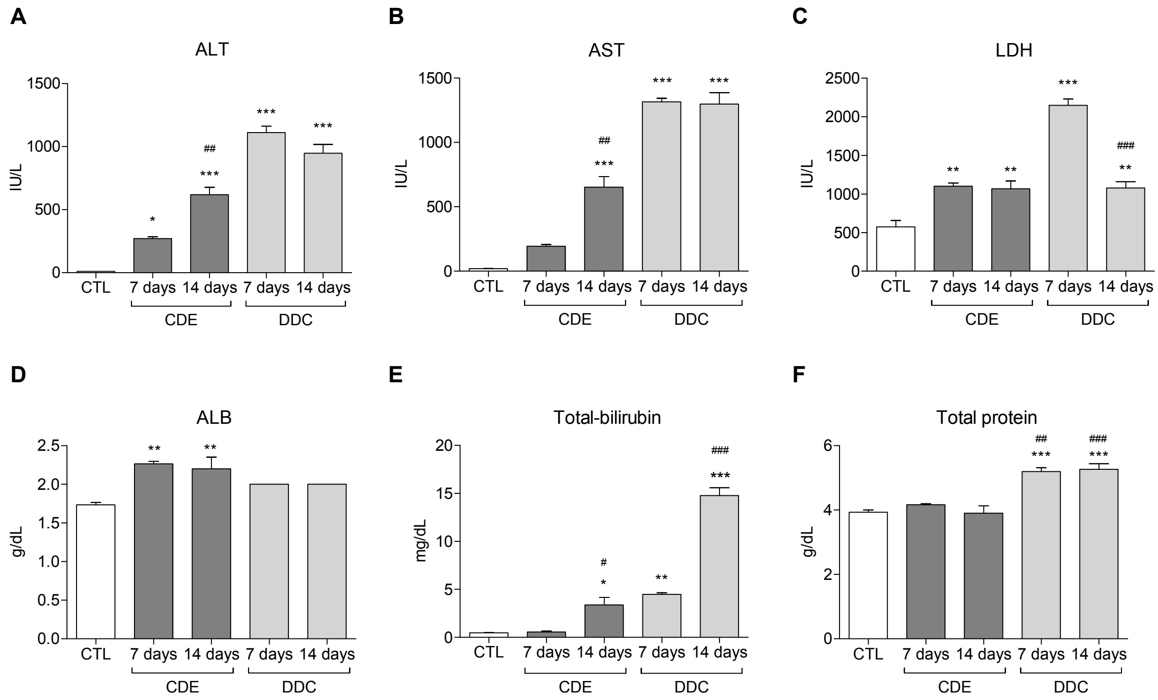

Figure S1. Quantification of clinical liver marker levels in serum of CDE- and DDC-treated mice. Mice received a CDE or DDC diet to induce liver injury during a time course of 7 and 14 days. Sera were collected and analyzed for determining levels of ALT (**A**), AST (**B**), LDH (**C**), ALB (**D**), total-bilirubin (**E**) and total protein (**F**). In the manuscript, serum parameters are mentioned as "fold increase" compared to control mice. All data are mean (+/-SEM) for n=5/group. *p<0.05, **p<0.01, and ***p<0.001 compared to controls. $^{\#}$p<0.05, $^{\#\#}$p<0.01, and $^{\#\#\#}$p<0.001 compared to treated groups (DDC or CDE). AST: aspartate aminotransferase; ALT: alanine transaminase and LDH: lactate dehydrogenase; ALB: albumin.

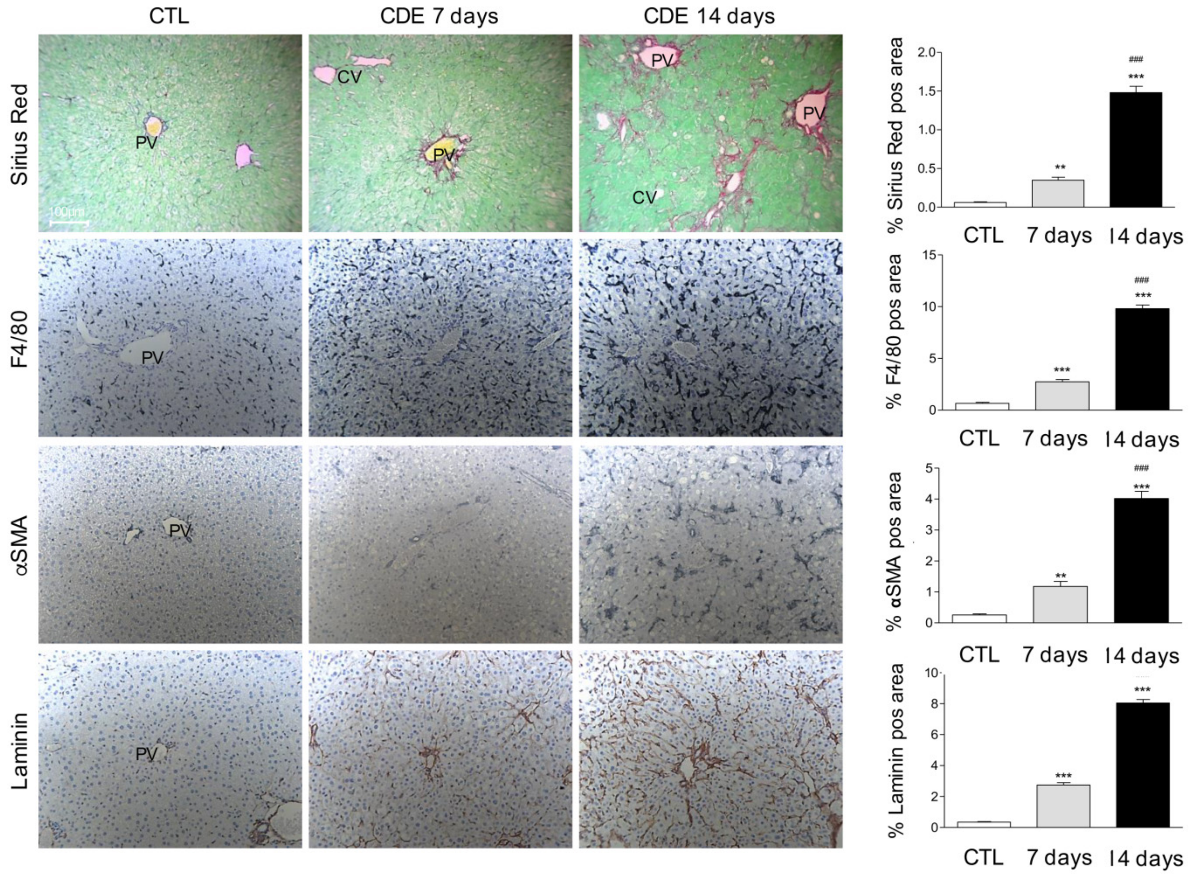

Figure S2. Illustration of the DR induced by the CDE dietary regime. Liver sections of control animals and mice that were subjected to a 7- or 14- day CDE-diet were stained with Sirius red, F4/80, αSMA and Laminin. The pictures display representative photomicrograph. All images were taken in 10x original magnification and then analyzed and quantified using ImageJ software. All data are mean (+/-SEM) for n=5/group. **p<0.01, and ***p<0.001 difference versus CTL. $^{###}$p<0.001 difference versus treated groups (CDE). PV: Portal vein; CV: Central vein.

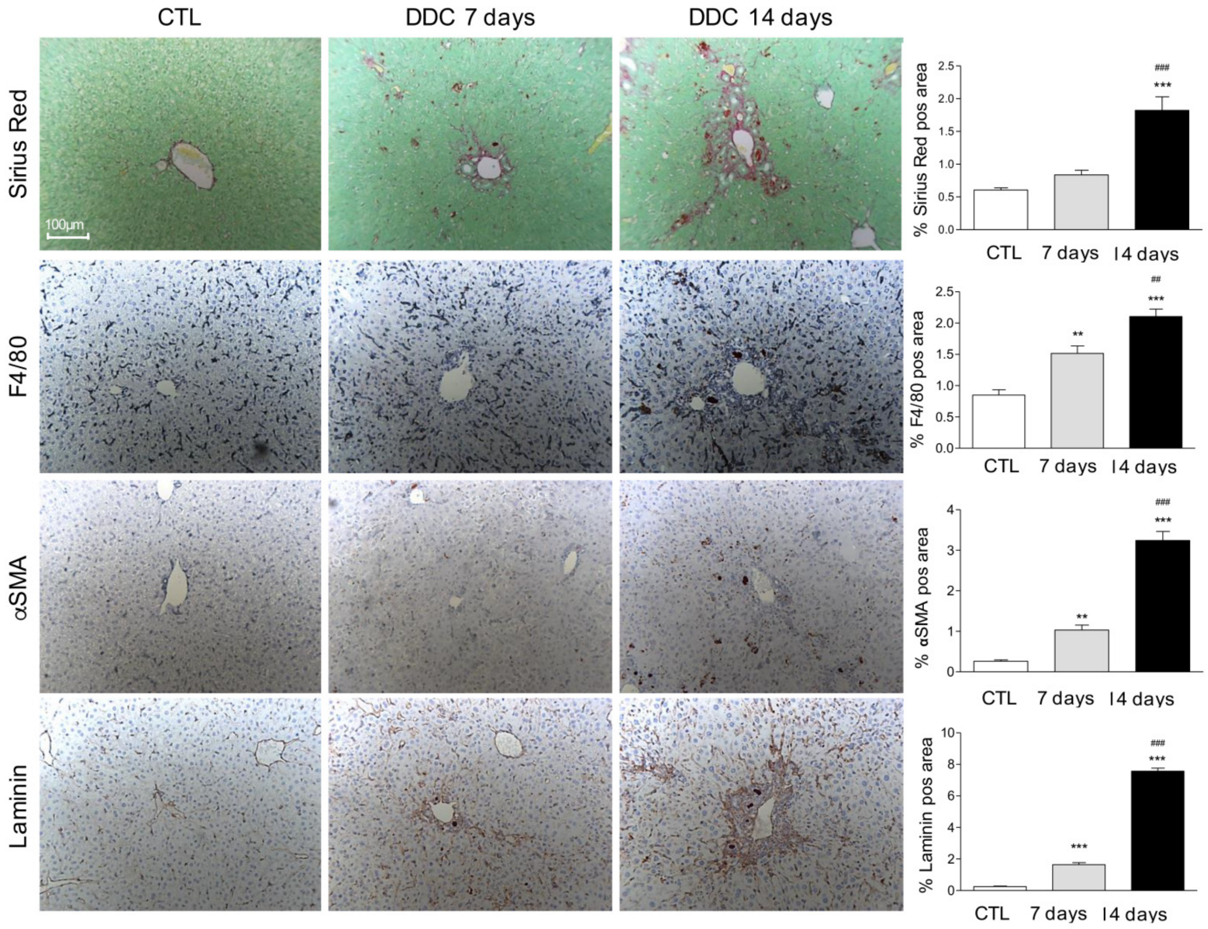

Figure S3. Illustration of the DR induced by DDC dietary regime. Liver sections of control animals and mice that were subjected to a 7 or 14 day DDC-diet were stained with Sirius red, F4/80, αSMA and Laminin. The pictures display representative photomicrograph. All images were taken in 10x original magnification and then analyzed and quantified by ImageJ software. All data are mean (+/-SEM) for n=5/group and the analysis of statistics is done with GraphPad Prism software. **$p<0.01$, and ***$p<0.001$ difference versus CTL. ###$p<0.001$ difference versus treated groups (DDC). PV: Portal vein; CV: Central vein.

A

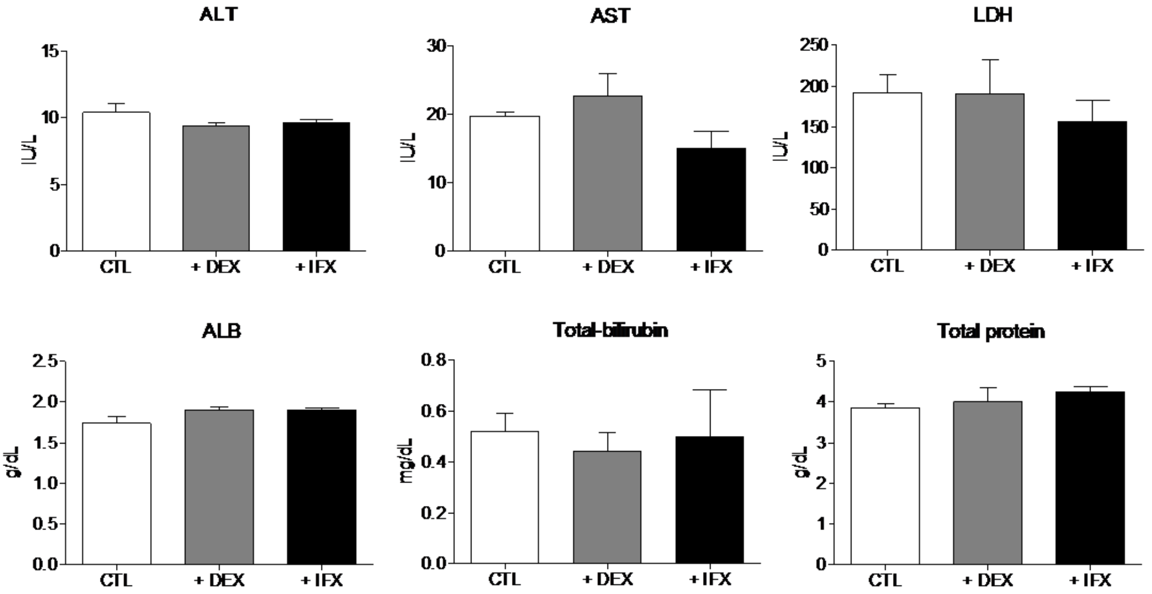

B

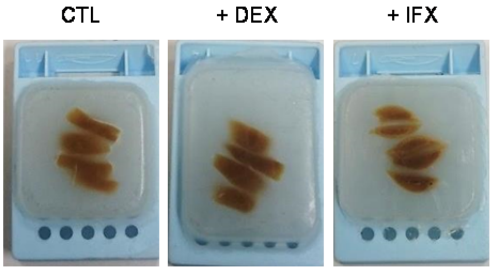

Figure S4. Administration of DEX or IFX in healthy mice has no impact on the levels of biochemical parameters and color of the livers. Healthy mice received by *i.p.* DEX or IFX in the same conditions described in M&M to examine a potential influence of these drugs on liver toxicity and biochemical parameters that reflect the well-being of the organ under these conditions. One week after the first injection, mice were sacrificed. (**A**) Sera were collected and analyzed for determining levels of ALT, AST, LDH, ALB, total-bilirubin and total protein. All data are mean (+/-SEM) for n=5/group. No significant difference was noticed. (**B**) Liver paraffin-embedding blocks are shown to illustrate the color of the hepatic tissues. Neither DEX nor IFX induce toxicity in the organ since all sera parameters were statistically normal and they have no direct influence on the color or texture of the liver.

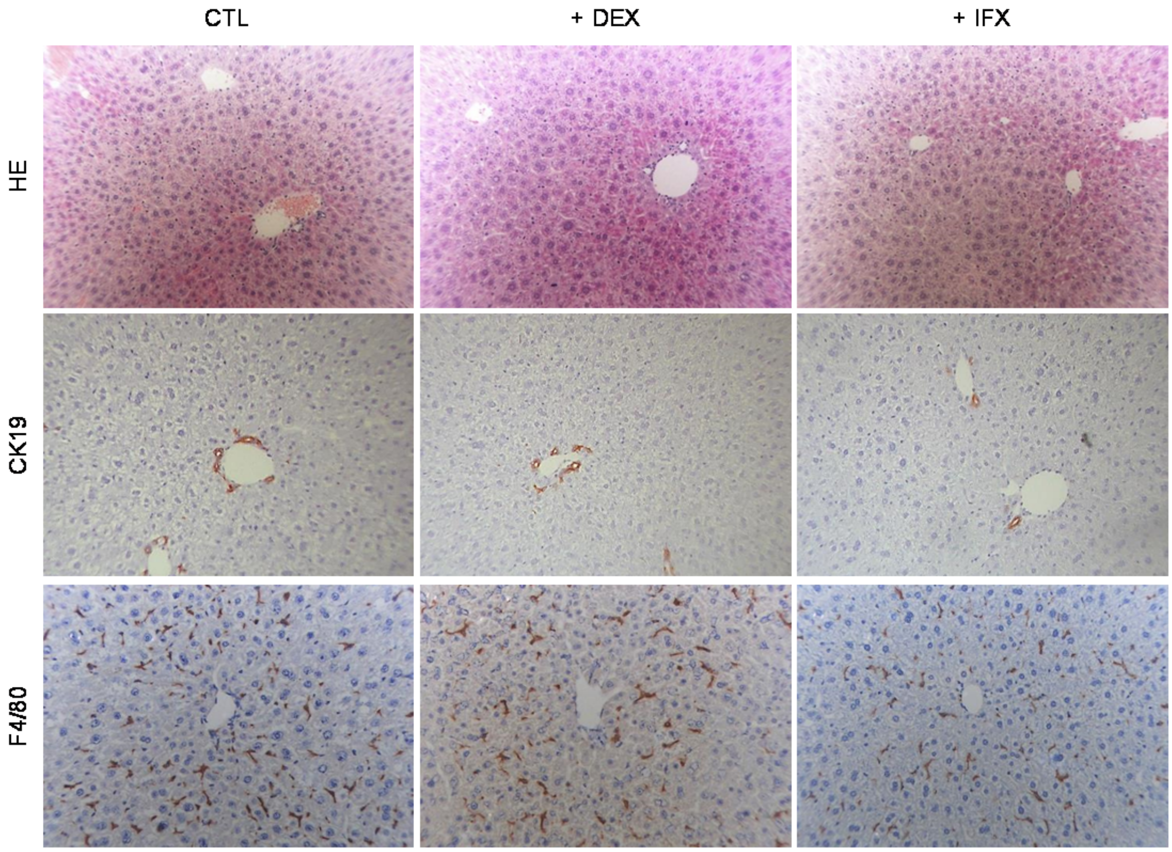

Figure S5. Administration of DEX and IFX in healthy mice has no impact on architecture of the liver and does not change the BECs and F4/80$^+$ cells. Healthy mice received DEX or IFX by *i.p.*injections in the same conditions described in M&M and were sacrificed 1 week later. Liver sections were then stained with eosin, CK19 and F4/80 antibodies and counterstained with hematoxylin. Magnification of the photographs is x20.

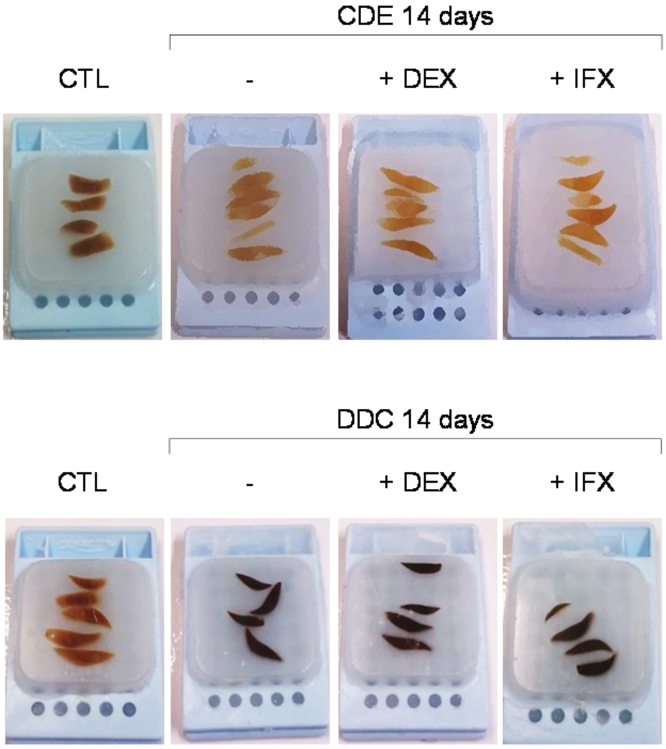

Figure S6. Administration of DEX and IFX in CDE- and DDC-treated animals has no effect on the color of the livers. Healthy and CDE- and DDC-treated mice received or not by *i.p.* DEX or IFX in the same conditions described in M&M and were sacrificed 1 week later. Half of the left lobes were collected, cut in slides and were proceeded into formalin bath for paraffin-embedding. The photographs illustrate the reddish color of the organ in healthy mice, while upon CDE or DDC treatments respectively, the livers turn pale or become darker due to porphyrin accumulation. Injections of DEX or IFX do not change the color or of the livers.

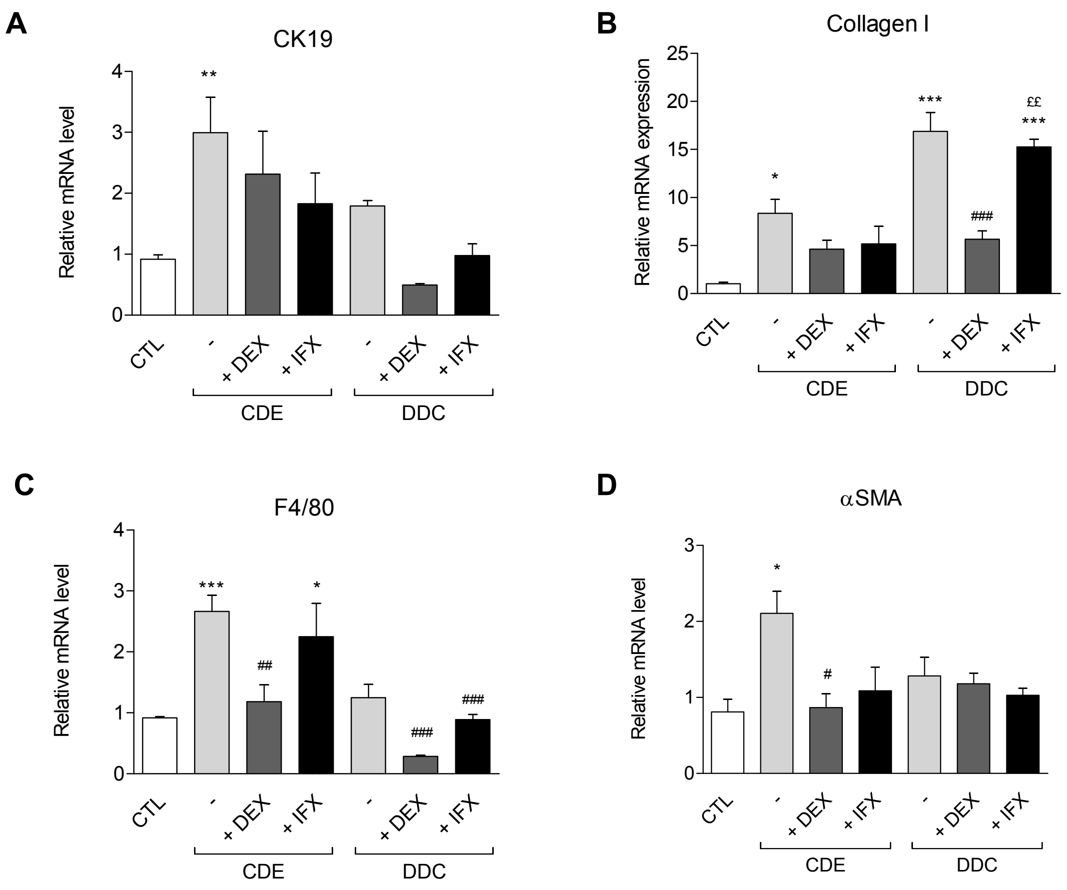

Figure S7. DEX and IFX reduce fibrogenesis in CDE- and DDC-treated livers. Livers were extracted out of the corresponding treated animals, and mRNAs were purified. PCR was then performed to detect the relative mRNA level for **A)** CK19, **B)** Collagen-1a1, **C)** F4/80 and **D)** αSMA. All data are mean for n=5/group. *p<0.05, **p<0.01, and ***p<0.001 difference versus CTL. #p<0.05, ## p<0.01, and ###p<0.001 difference versus treated groups (DDC or CDE). $^{££}$p<0.01 difference versus co-treated groups (+/-DEX or +/-IFX).

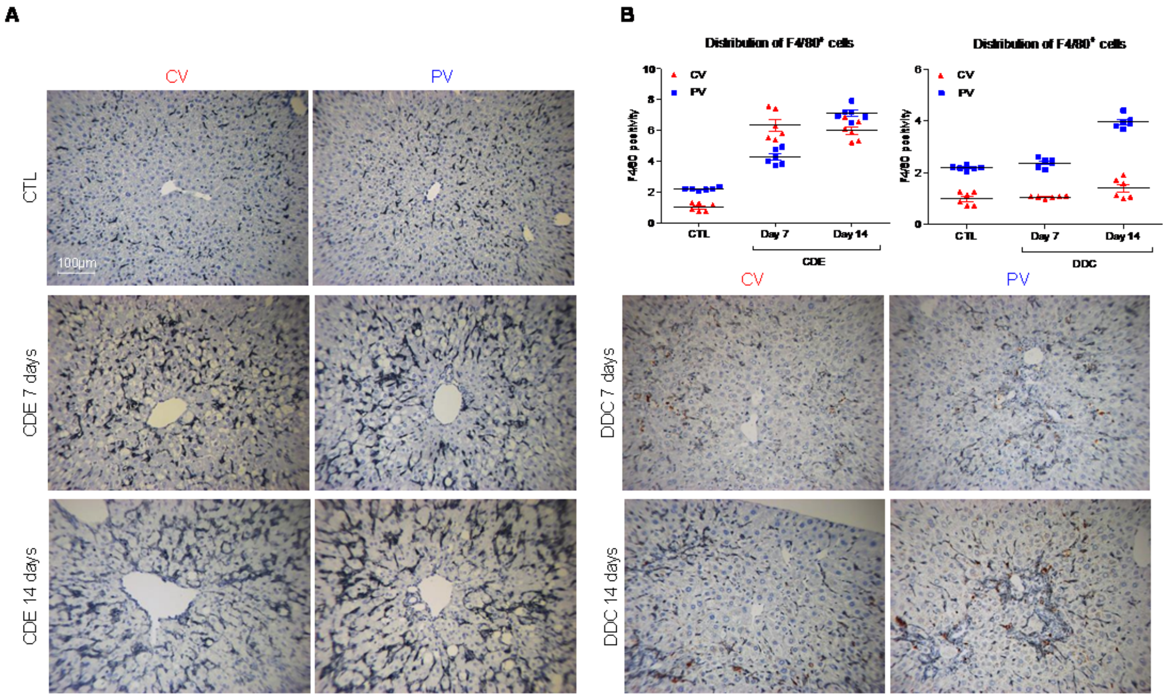

Figure S8. Spatial distribution of F4/80-positive cells in mice treated with CDE and DDC diets. (**A**) Healthy mice or mice subjected to a period of 14 day CDE - or DDC-diet in presence or absence of DEX or IFX were analyzed for spatial distribution of F4/80^{+} cells. Livers sections were stained with F4/80 to visualize the Kupffer cells and counterstained with hematoxylin. (**B**) Independent quantification of F4/80 positivity from both portal vein (PV) and central vein (CV) are illustrated in both CDE and DDC diets.

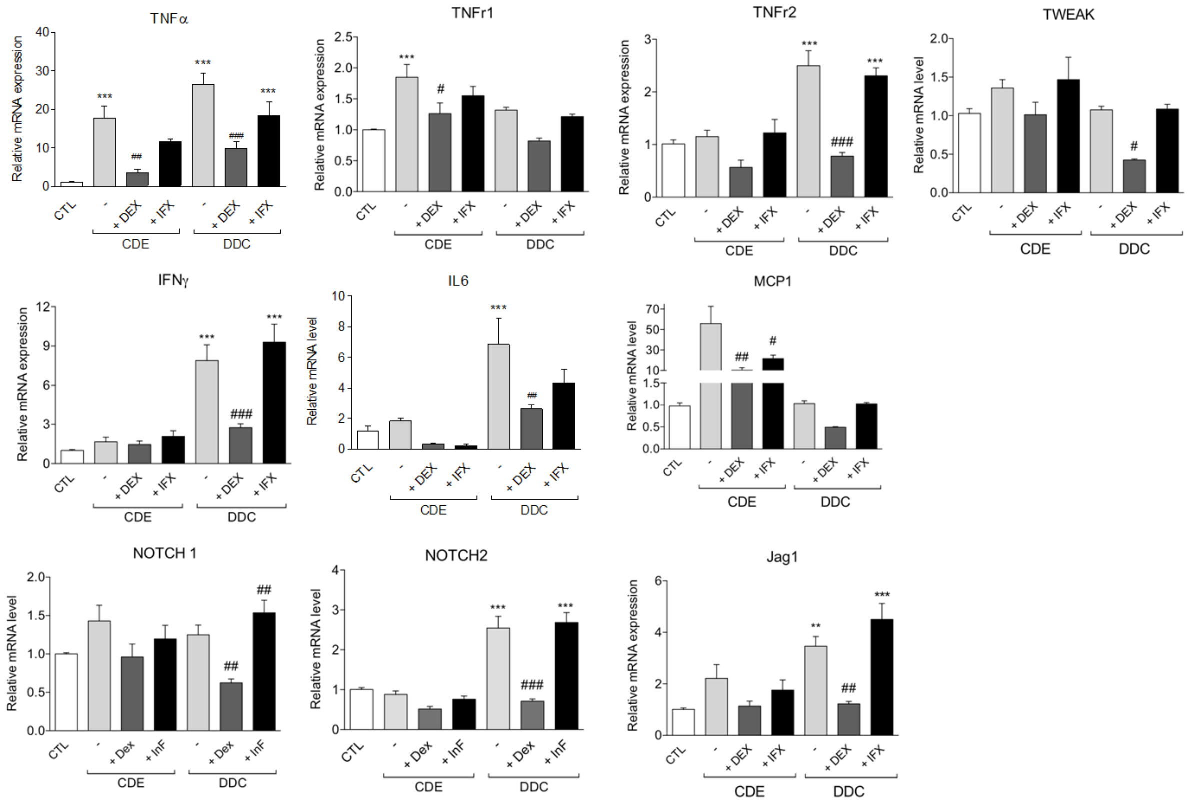

Figure S9. DEX and IFX reduce expression of key cytokines in CDE and DDC induced liver injuries. mRNA levels of Tnfα, Il6, Mcp1, Tnfr1/2, Tweak, Ifnγ, Notch1/2 and Jag1 were determined in livers at the end of the 14 day treatment of mice with CDE or DDC diet, with or without co-treatment with DEX and IFX. **p<0.01, and ***p<0.001 difference versus CTL. #p<0.05, ## p<0.01, and ###p<0.001 difference versus treated groups (DDC or CDE).

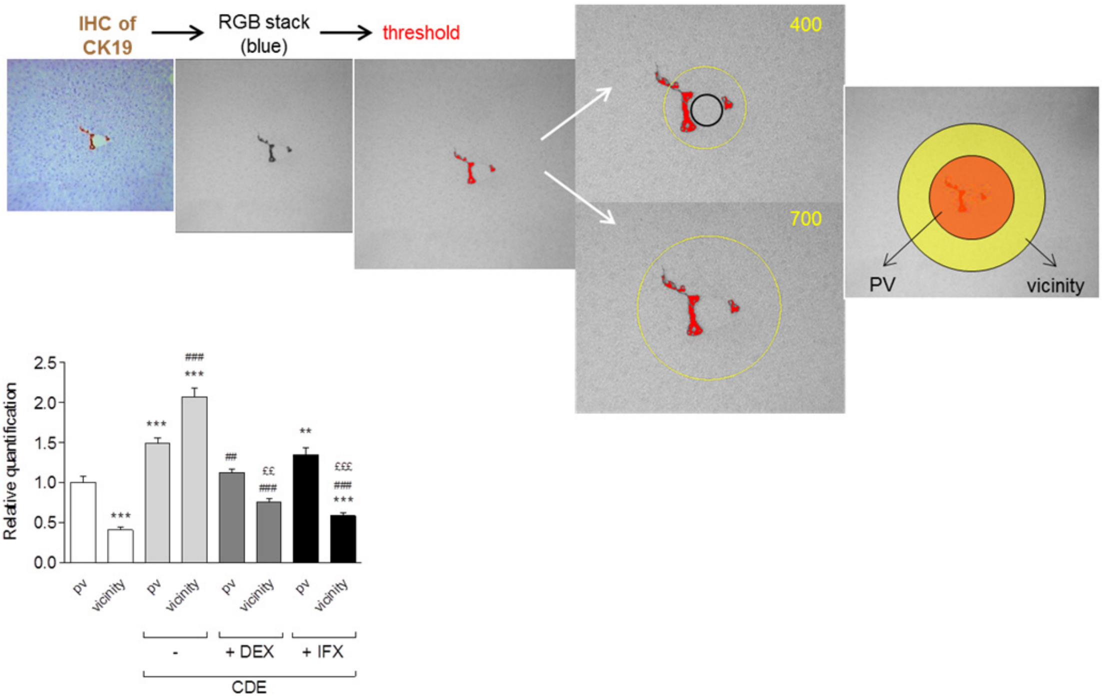

Figure S10. Relative quantification and distribution of the CK19$^+$-area close to PV or to its vicinity upon CDE treatment in presence or absence of DEX and IFX. All data are mean for n=5/group. **p<0.01, and ***p<0.001 difference versus CTL. ## p<0.01, and ###p<0.001 difference versus treated groups (DDC or CDE). $^{££}$p<0.01, and $^{£££}$p<0.001 difference versus co-treated groups (+/-DEX or +/-IFX).

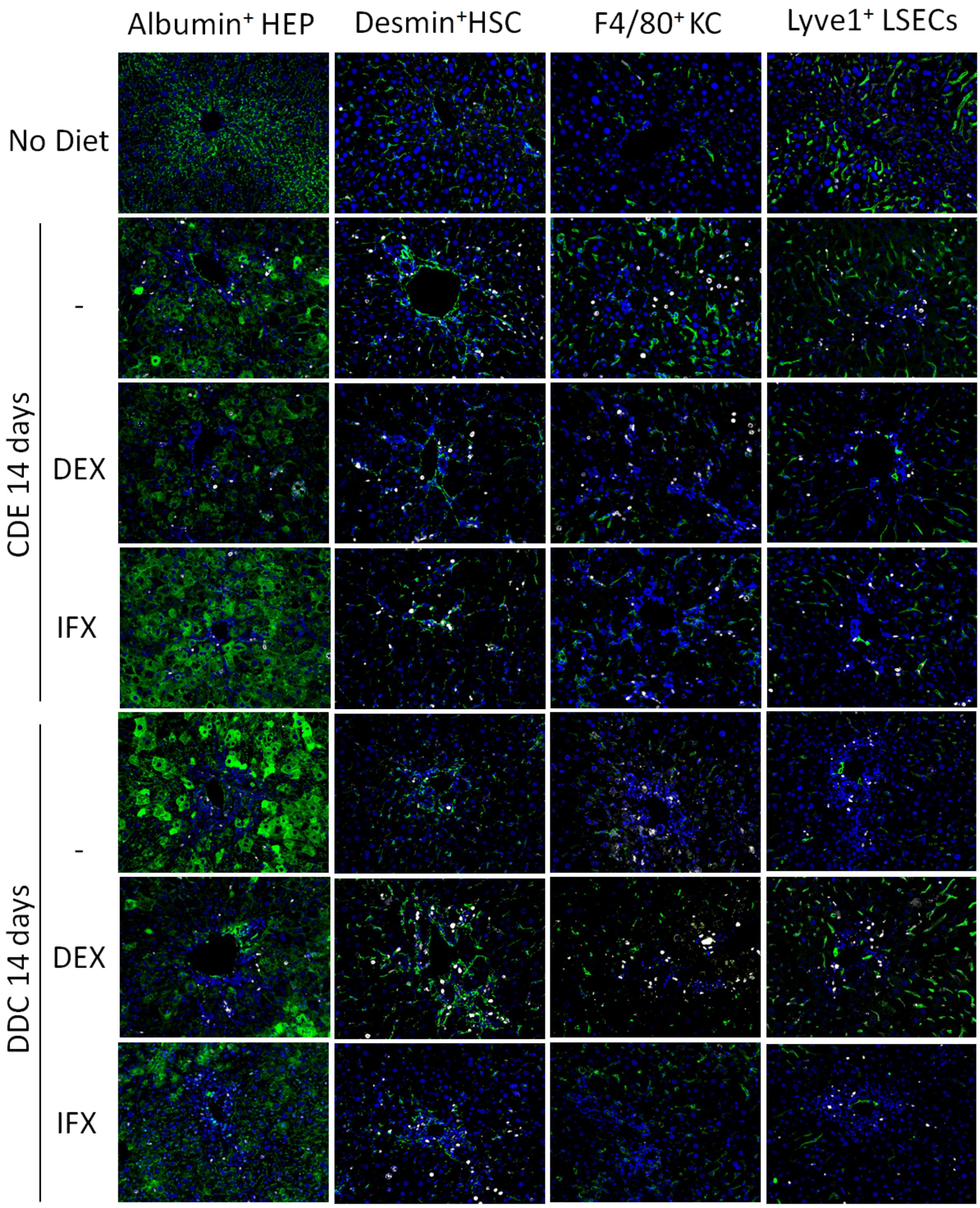

Figure S11. Influence of CDE and DDC, and DEX and IFX co-treatment on proliferative status of hepatic cells. Immunofluorescence stainings of nuclei (blue), Ki-67 positive cells (white) and a cell-specific marker (green) for hepatocytes (HEP, Albumin), hepatic stellate cells (HSC, Desmin), kupffer cells (KC, F4/80) and liver sinusoidal endothelial cells (LSEC, Lyve1) were determined in livers at the end of the 14 day treatment in healthy mice or mice subjected to a period of 14 day CDE - or DDC-diet in presence or absence of DEX or IFX. Quantification of these images and the images for the CK19^{+}Ki-67^{+} staining are given in Figure 7 of the main text.

Table S1. Conditions of immunohistochemistry/immunofluorescence used for the study

Antigen	Antigen retrieval	Dilution I^{ary} Abs (incubation)	Application
**F4/80**	Proteinase K (8min @RT)	1/200 (1hr30)	IHC
**CK19**	Proteinase K (8min @RT)	1/200 (1hr30)	IHC/IF
**Ki67**	Citrate buffer (20min @ 99°C; 20min @RT)	1/200 (o/n)	IHC/IF
**Laminin**	Proteinase K (8min @RT)	1/5000 (1hr)	IHC
**αSMA**	Citrate buffer (20min @ 99°C; 20min @RT)	1/600 (o/n)	IHC
**F4/80**	Citrate buffer (20min @ 99°C; 20min @RT)	1/200	IF
**Lyve 1**	Citrate buffer (20min @ 99°C; 20min @RT)	1/200	IF
**Desmin**	Citrate buffer (20min @ 99°C; 20min @RT)	1/200	IF
**Albumin**	Citrate buffer (20min @ 99°C; 20min @RT)	1/200	IF

Table S2. List of antibodies used for the experiments

Antigen	Species	Manufacturers (reference)	Clones
**F4/80**	Monoclonal rat antibody	Serotec (MCA497GA)	Cl:A3-1
**CK19**	Monoclonal rat antibody	DSHB	TROMA-III
**Ki-67**	Monoclonal rabbit antibody	Thermo Scientific	SP6
**Laminin**	Polyclonal rabbit antibody	Dako (Z0097)	n.m
**αSMA**	Monoclonal rabbit antibody	AbCam (ab32575)	E184
**Alexa Fluor 488**	Donkey Anti-rabbit IgG (H+L)	Thermo Scientific (A11006)	
**Lyve 1**	Polyclonal goat antibody	R&D systems (AF2125)	
**Desmin**	Polyclonal rabbit antibody	Thermo Scientific (RB-9014-P1)	
**Albumin**	Polyclonal goat antibody	Novo Biologicals (NB600-41532)	
**Ki-67**	Polyclonal rat antibody		
**Alexa Fluor 594**	Donkey Anti-rabbit IgG (H+L)	Thermo Scientific (A21207)	
**Alexa Fluor 594**	Donkey Anti-rat IgG (H+L)	Thermo Scientific (A21209)	
**Alexa Fluor 488**	Donkey Anti-rat IgG (H+L)	Thermo Scientific (A21208)	
**Alexa Fluor 488**	Donkey Anti-goat IgG (H+L)	Thermo Scientific (A11055)	

Table S3. Oligonucleotides used for quantitative RT-PCR

Gene	NM	Primer sequences (Fwd./Rv.)
**acta2 (αSMA)**	007392.3	CCAGCACCATGAAGATCAAG<br>TGGAAGGTAGACAGCGAAGC
**ck19**	008471.2	ATGAGATCATGGCCGAGAAG<br>GGTGTTCAGCTCCTCAATCC
**tnfα**	013693.1	TCTTCTCATTCCTGCTTGTGG<br>GGTCTGGGCCATAGAACTGA
**mcp1**	011333.3	CATCCACGTGTTGGCTCA<br>GATCATCTTGCTGGTGAATGAGT
**f4/80**	010130.4	CCTGGACGAATCCTGTGAAG<br>GGTGGGACCACAGAGAGTTG
**il6**	031168.1	GCTACCAAACTGGATATAATCAGGA<br>CCAGGTAGCTATGGTACTCCAGAA
**gapdh**	008084.2	CCTGCTTCACCACCTTCTTG<br>TGTCCGTCGTGGATCTGAC
**collagen 1 a1**	007742.3	ACCTAAGGGTACCGCTGGA<br>TCCAGCTTCTCCATCTTTGC
**tnfr1**	011609.4	CCGGGAGAAGAGGGATAGCTT<br>TCGGACAGTCACTCACCAAGT
**tnfr2**	011609.4	ACACCCTACAAACCGGAACC<br>AGCCTTCCTGTCATAGTATTCCT
**notch1**	008714.3	TGCAACTGTCCTCTGCCATA<br>GTAGCACATGGGGCCAAC
**notch2**	010928.2	TTACCTACCACAACGGCACA<br>GGCAATATTCTCCCAAGAAGC
**ifnγ**	212864.1	ATCTGGAGGAACTGGCAAAA<br>TTCAAGACTTCAAAGAGTCTGAGG
**tweak**	011614.3	GCCCATTATGAGGTTCATCC<br>TCACTGTCCCATCCACACC
**jag1**	013822.5	ATGCAGAACGTGAATGGAGAG<br>GCGGGACTGATACTCCTTGAG
